# Neuroprotective Effects of Different Modalities of Acupuncture on Traumatic Spinal Cord Injury in Rats

**DOI:** 10.1155/2014/431580

**Published:** 2014-04-03

**Authors:** Song-he Jiang, Wen-zhan Tu, En-miao Zou, Jie Hu, Sai Wang, Jiang-ru Li, Wan-sheng Wang, Rong He, Rui-dong Cheng, Wei-jing Liao

**Affiliations:** ^1^Department of Physical Medicine and Rehabilitation, Zhongnan Hospital of Wuhan University, Donghu Road, Wuhan, Hubei, China; ^2^Department of Physical Medicine and Rehabilitation, The Second Affiliated Hospital of Wenzhou Medical University, Xueyuan Road, Wenzhou, Zhejiang, China; ^3^Department of Rehabilitation Medicine, Zhejiang Hospital, Lingyin Road, Hangzhou, Zhejiang, China; ^4^Department of Rehabilitation Medicine, Zhejiang Provincial People's Hospital, Shangtang Road, Hangzhou, Zhejiang, China

## Abstract

Spinal cord injury (SCI) can induce a series of histological, biochemical, and functional changes. Acupuncture is commonly used for SCI patients. Using male rats of spinal cord injury with the New York University (NYU) Impactor, we investigated the response of electroacupuncture (EA), manual acupuncture (MA), and transcutaneous acupoint electrical stimulation (TAES) at Shuigou (DU26) and Fengfu (DU16) acupoints to understand the effects and mechanisms of acupuncture in neuroprotection and neuronal function recovery after SCI. Histological study showed a restored neural morphology and an increase in the quantity of neurons after EA, MA, and TAES administrations. Acupuncture's antioxidation effects were demonstrated by alleviation of the post-SCI superoxide dismutase (SOD) activity increase and malondialdehyde (MDA) level decrease. The anti-inflammation effect of acupuncture was shown as the reduced expression of inflammatory cytokines including interleukin-1**β** (IL-1**β**), interleukin-6 (IL-6), and tumor necrosis factor-**α** (TNF-**α**) when SCI was treated. And the antiapoptosis role was approved by TUNEL staining. Our data confirmed that the role of acupuncture in neuroprotection and dorsal neuronal function recovery after rat SCI, especially, EA stimulating at Shuigou (DU26) and Fengfu (DU16) can greatly promote neuronal function recovery, which may result from antioxidation, anti-inflammation, and antiapoptosis effects of acupuncture.

## 1. Introduction

Spinal cord injury (SCI) is a fatal event which leads to physical, psychological, and social impact on individuals, family, and society around the world [1]. However, at present, there still is lack of effective treatments for spinal cord injuries [2, 3]. Some studies have demonstrated that the treatment during the time period between the primary and secondary injury has the potential to either prevent or to reduce the final neurological deficits [4]. Acupuncture is a therapeutic technique used in traditional Chinese medicine and involves puncturing the skin with thin sterile needles at well-defined acupuncture points which may be excitable muscle/skin-nerve complexes with a high density of nerve endings. The needles are primarily stimulated manually or electrically. In manual acupuncture (MA) the needles are twisted back and forth. In addition to traditional manual acupuncture, new acupuncture modalities, such as electroacupuncture (EA) and transcutaneous acupoint electrical stimulation (TAES), are gaining in popularity. In EA, a stimulating current at various parameters is applied to acupoints through the needles [5]. Meanwhile, in TAES, electrical pulses are delivered on the skin of the acupoints via electrode [6]. Compared to MA, EA is more effective in pain relief [7] in both humans and animal models, and TAES has been shown to be as effective as EA in analgesia [8]. Previous studies showed that applications of EA for the treatment of SCI had been proven to contribute towards neurologic and functional recoveries in SCI [9–11]. It is reasonable to study whether different modalities of acupuncture could induce different responses.

Shuigou (DU26) and Fengfu (DU16) are important acupoints located in the DU Vessel which runs posterior along the interior of the spinal column and relates to the function of spinal cord according to the traditional theory in traditional Chinese medicine [12]. Previous studies showed that acupuncture applied at both Shuigou (DU26) and Yanglingquan (GB34) acupoints simultaneously exerted a neuroprotection which would be partly mediated via inhibition of inflammation and microglial activation; furthermore, acupuncture alleviated neuropathic pain induced by SCI [13, 14]. However, there were no prior experiments of EA applied at DU26 and DU16 simultaneously after SCI.

The aim of the study was to evaluate the efficacy on the two acupoints (DU26 and DU16) induced by three popularly utilized acupuncture modalities, namely, MA, EA, and TAES, by comparing their antioxidation, anti-inflammation, and antiapoptosis effects and the reduction of apoptotic cell death of neurons, thereby leading to improved neuronal function recovery after SCI.

## 2. Experimental Procedures 

### 2.1. Animal and Experimental Groups

All experiments were approved by the Institutional Animal Care and Use Committee of Wenzhou Medical University. All the 110 rats (Sprague-Dawley, male, 180 to 220 g) were randomly assigned into five groups. The sham group (*n* = 22), received only a laminectomy. The remaining four groups underwent spinal cord injury at the T10 spinal segment impactor. The control group (*n* = 22) received no treatment following spinal cord injury, the EA group (*n* = 22) received EA treatment at DU26 and DU16 acupoints, and the MA (*n* = 22) group received MA treatment at the same acupoints, while the TAES group (*n* = 22) received transcutaneous electrical stimulation at the same acupoints. All of the animals were housed in separated cages with free access to food and water. Room temperature was set at 25 ± 3°C.

### 2.2. Spinal Cord Injury

Moderate spinal cord injury was induced using the NYU Impactor. The rats were anesthetized with chloral hydrate (500 mg/kg), and a laminectomy was performed at the T10 level to expose the cord beneath without disrupting the dura. The spinous processes of T9 and T11 were then clamped to stabilize the spine, and the exposed dorsal surface of the cord was subjected to contusion injury (10 g × 25 mm) using the NYU Impactor [15, 16] with the exception of the sham group. All animals models included in the study met the following criteria: spinal cord ischemia and edema around the wound, formation of tail sway reflex, flicking of both body and legs, and appearance of sluggish paralysis.

The wound was covered with cotton soaked in saline to avoid direct contact of the spinal cord with air. The Institutional Animal Care and Use Committee of Wenzhou Medical University approved all surgical interventions and postoperative animal care.

### 2.3. Acupuncture Application

**Figure 1:**
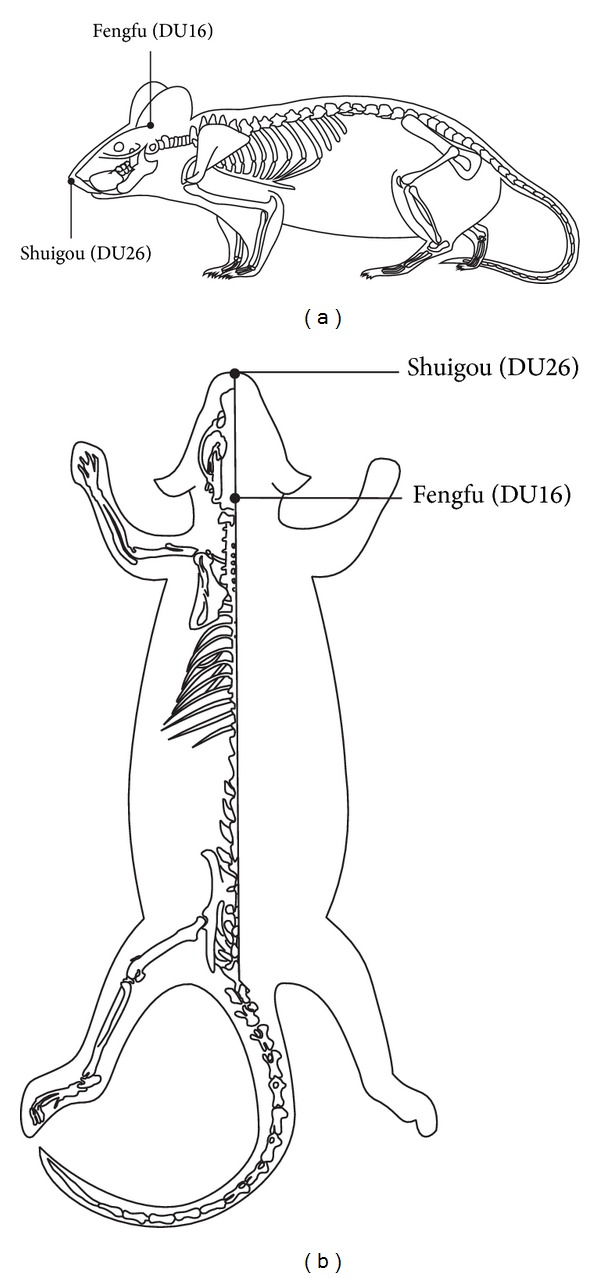
Drawings (a) and (b) show where acupuncture was applied.

**Figure 2:**
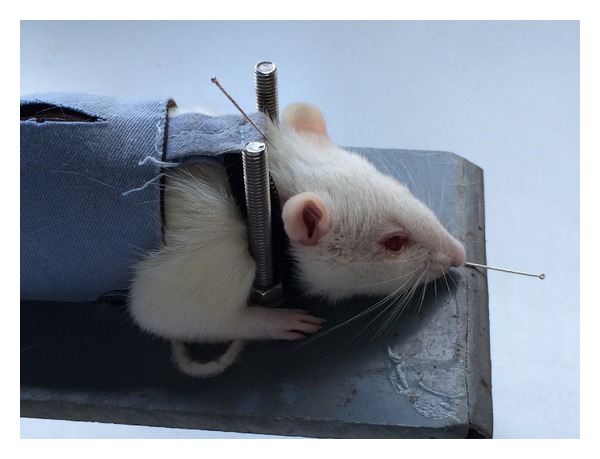
Rats immobilization apparatus for acupuncture.

DU26 and DU16 acupoints were utilized during EA, MA, or TAES treatment. The DU26 acupoint is located in the anterior midline and in the depression below the nose. DU16 is located in the posterior midline and in the depression below the spinous process of the second cervical vertebra in prone position (Figure 1). Rats were kept in an immobilization apparatus designed by our laboratory (Chinese Patent Application Number: 201110021482.5, State Intellectual Property Office) without anesthesia. The system is designed to be both convenient for acupuncture research and comfortable for experimental rats to reduce stress (Figure 2).

In EA or MA group, one pair of stainless steel needles of 0.25 mm in diameter were inserted into the DU26 (a depth of 5 mm) and DU16 (a depth of 7.5 mm). In the EA group, the needles were connected to the output terminals of an EA apparatus (HANS-200E, Jisheng Medical Instruments) and stimulated by continuous wave of 2 Hz frequency and 0.2 mA intensity for 30 minutes. In MA group, the needles were turned at a rate of two spins per second for 10 seconds every ten minutes during a 30-minute period. In the TAES group, the two acupoints were patched with the same transcutaneous apparatus and stimulation parameters which were continuous wave, 2 Hz frequency and 1 mA intensity for 30 minutes. EA, MA, and TAES were administrated both at 2 hours and 8 hours of postsurgery.

### 2.4. HE Staining and Nissl Staining

At the 8th hour after injury surgery, we administrated the second time of EA, MA, and TAES treatments. Eight of 22 rats in each group were sacrificed at the 8.5th hour of postsurgery and perfused transcardially with 0.9% sodium chloride, and then with 4% paraformaldehyde in 1X phosphate buffered saline (4% PFA) for 30 min. Spinal cords were dissected out and kept in 4% PFA for postfix overnight. After dehydration, the spinal cords were embedded with paraffin, and serial coronal sections with thickness of 5 *μ*m were obtained. To assess the histopathologic change, each one of five sections obtained was subjected to HE and Nissl staining.

### 2.5. TUNEL Staining

To detect apoptosis, each one of five coronal sections obtained in the above experimental procedure was further subjected to terminal deoxynucleotidyl transferase mediated dUTP nick end labeling (TUNEL) staining. Apoptotic cells were characterized by dark brown staining of nucleus and nuclear membrane. Quantitation was performed by counting the quantity of positive cells in five randomly chosen fields within each slide at 400x with an Olympus (CH30, Japan) optic microscope. The index of apoptosis was calculated as the ratio of overall apoptotic cells.

### 2.6. Biochemical Analysis

Additional 6 of 22 rats in each group were sacrificed at the 8.5th hour of postsurgery. Tissues of the injured spinal cord were dissected out and homogenized at a concentration of 100 g/L, centrifuged at 3500 rpm for 20 min at −10°C, and stored at −20°C. To evaluate the effect of acupuncture, biochemical kits (Beyotime Institute of Biotechnology, China) were used to measure lipid peroxidation end product malondialdehyde (MDA) and biochemical kits (Dojindo Molecular Technologies, Inc., Japan) were used to measure superoxide dismutase (SOD). All procedures completely complied with the manufacturer's instructions. The assay of SOD activity was based on its ability to inhibit the oxidation of oxymine by superoxide anion produced from the xanthine-xanthine oxidase system. One unit of SOD activity was defined as the amount that reduced the absorbance at 550 nm by 50%.

### 2.7. Western Blotting

The remaining 8 of 22 rats in each group were deeply anesthetized with 4% chloral hydrate and sacrificed at the 8.5th hour of postsurgery. A 1 cm spinal cord centered at the injury epicenter was quickly dissected, and then the tissues were cut longitudinally along the middle line into two homogeneous parts to make sure each half contains the wound site. Half of the injured spinal cord sample was performed to investigate the expression of the IL-1*β*, IL-6, and TNF-*α* protein by Western blot analysis. Protein homogenates of spinal cord were prepared by rapid homogenization in 10 volumes of lysis buffer (2 mM EDTA, 10 mM EGTA, 0.4% NaF, and 20 mM Tris-HCl, pH 7.5). Samples were centrifuged at 17000 ×g for 1 h and the protein concentration of soluble materials was determined by the Coomassie G250 binding method. Protein lysates were prepared at 12 *μ*g/lane for each sample and then fractioned on 12% SDS-polyacrylamide gels. Electroblotting proteins were transferred to nitrocellulose membranes (Abcam Biotechnology, Inc.). The blots were then incubated with goat polyclonal antibody anti-IL-1*β* (1 : 500 R&D Biotechnology, Inc.), mouse monoclonal antibody anti-IL-6 (1 : 1000), and rabbit polyclonal antibody anti-TNF-*α* (1 : 2000; Abcam Biotechnology, Inc.). IL-1*β*, IL-6, and TNF-*α* band on the immunoblots were visualized using the enhanced chemiluminescence (ECL kit, Santa Cruz Biotechnology, Inc.). The IL-1*β*, IL-6, and TNF-*α* protein bands and *β*-actin bands were scanned using ChemiImager 5500 V2.03 software, and IDVs were calculated by Fluor Chen 2.0 software and normalized with that of *β*-actin.

### 2.8. Real-Time PCR

Using the TRIZOL Reagent (Invitrogen 15596-026), total RNA was isolated from the other half part of the 1 cm spinal cord fragment which was described in above procedure. cDNA synthesis using Revert Aid First Strand cDNA Synthesis Kit (Fermentas K1622) and RT-qPCR was carried out using specific primers, as previously described. For relative quantification, each gene of interest was first subjected to a serial dilution assay to determine the optimum detection range of Ct values, with a Ct threshold of 35 for undetectable levels of expression. Using 10 ng of reverse-transcribed total RNA, 20 pmol/mL of both sense and antisense primers, and the Fast SYBR Premix Ex TaqTM (TaKaRa code: DRR420A) in a final reaction volume of 20 *μ*L, the reactions were run on an ABI PRISM 7900 Fast Sequence Detection System instrument and software (Applied Biosystem) according to the manufacturer's protocol. The primers used for TNF-*α*, IL-1*β*, and IL-6 were synthesized by the Genotech (Daejeon, Korea). The sequences of the primers were 5′-CCC AGA CCC TCA CAC TCA GAT-3′ (sense) and 5′-TTG TCC CTT GAA GAG AAC CTG-3′ (antisense) for TNF-*α*; 5′-GCAGCT ACC TAT GTC TTG CCC GTG-3′ (sense) and 5′-GTC GTT GCT TGTCTC TCC TTG TA-3′ for IL-1*β*; 5′-AAG TTT CTC TCC GCA AGA TAC TTCCAG CCA-3′ (sense) and 5′-AGG CAA ATT TCC TGG TTA TAT CCA GTT T-3′ (antisense) for IL-6; 5′-TCC CTC AAG ATT GTC AGC AA-3′ (sense) and 5′-AGA TCC ACA ACGGAT ACA TT-3′ (antisense) for GAPDH, which was used as an internal control.

### 2.9. Data Analysis

The data were analyzed using one-way ANOVA. If equal variances were found, Fisher's least significant difference test was performed. Otherwise, the Kruskal-Wallis Test and Dunnett's T3 were used. The statistical significance level was set at *P* < 0.05.

## 3. Results

### 3.1. Different Effects of Electrical, Manual Acupuncture and Transcutaneous Acupoint Electric Stimulation on Histological Changes after SCI

**Figure 3:**
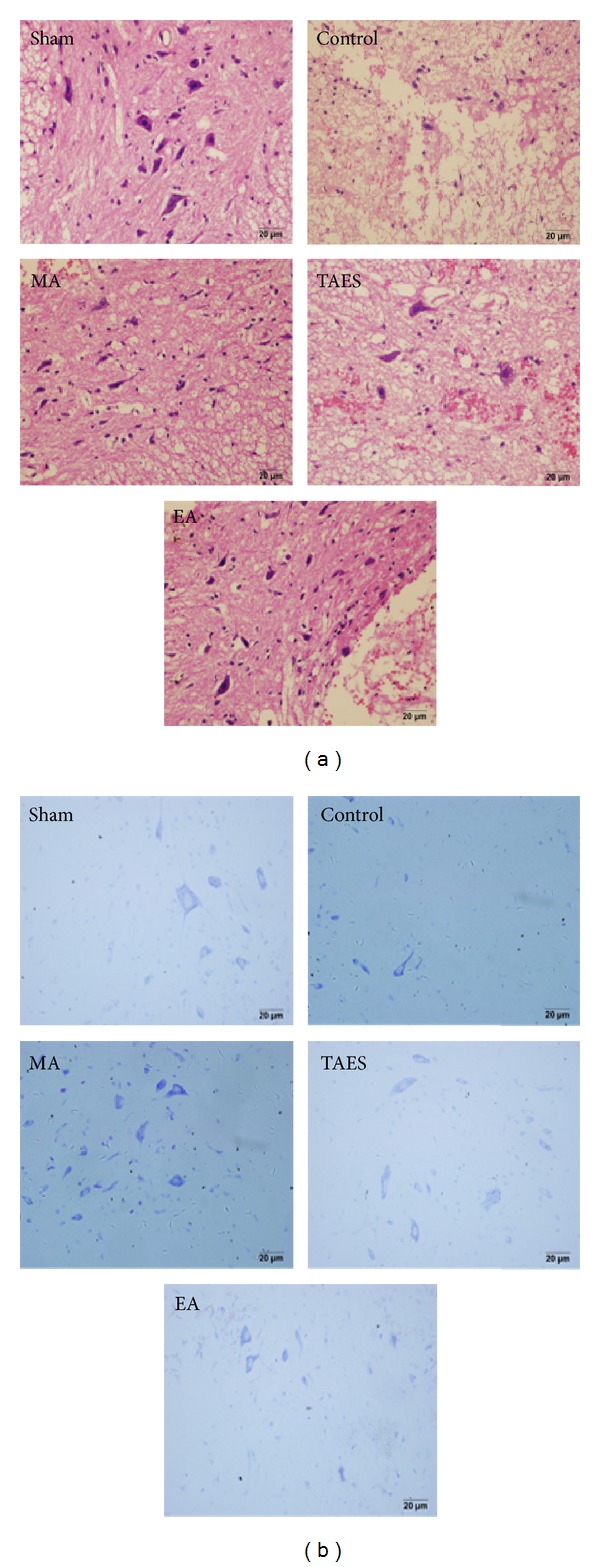
HE staining and Nissl staining. (a) HE staining showed normal neural morphology in the sham group. In the control group, impacted spinal cord exhibited typical necrosis showing as broad hemorrhage, edema, and neuronal apoptosis with condensed nuclei. All in the EA, MA, and TAES group neurons displayed normal morphology with clear boundaries. Compared with the control group, hemorrhage and edema occurred in the EA, MA, and TAES groups. (b) In the sham group, neurons exhibited a large amount of densely stained toluidine blue granules in the cytoplasm. However, in the control group, the Nissl bodies dramatically decreased or even disappeared in the neurons. In the EA, MA, and TAES groups the quantity of Nissl bodies was restored compared with that of the control group and displayed patch morphology.

To evaluate the protective effects, we compared histopathological alterations in spinal cord after stimulation of EA, MA, and TAES at DU26 and DU16. HE staining (Figure 3(a)) showed that spinal cord in the sham group had integrated infrastructures and clear boundary between gray and white matters. Blood vessels and central canal also exhibited normal morphology. No neuronal apoptosis was observed in the sham group. Boundaries in the control group became obscured and broad hemorrhages were found in both gray and white matters. Patches of necrosis were seen in the gray matter as well as liquefaction surrounding the damaged tissues. In addition, gaps between cells and blood vessels became relatively larger. A portion of neurons were found with condensed nuclei and darkly red stained cytoplasm. A number of apoptotic bodies were also noted. The extent of neuronal damages in the EA, MA, and TAES groups was between that of the sham group and that of the control group. Spinal cord lacked clear infrastructures and cellular boundaries, while the degree of hemorrhage, necrosis, and peripheral tissue edema was mild compared with the control group. However, dim histological staining indicated blurring structures in some remaining neurons.

Nissl staining (Figure 3(b)) showed that neurons in the sham group displayed integrative and granular-like morphology. The plasma was densely stained with toluidine blue, indicating active supply of neuronal nutrients and energy synthesis. However, in the control group the quantity of neurons was remarkably lower and neurons appeared with irregular morphologies. Intracellular toluidine blue staining was also significantly reduced, dimly spread out over a small region. SCI induced neuronal necrosis and apoptosis which lead to neuronal loss. Remaining neurons had difficulty in energy synthesis which resulted in neuronal dysfunction. In the three treatment groups, we found active neurons, though the quantity was reduced. However, tissue morphology was relevantly maintained with lighter staining in the cytoplasm and granular-like morphology.

### 3.2. Effects of Electrical, Manual Acupuncture and Transcutaneous Acupoint Electric Stimulation on the Inhibition of Apoptotic Cell Death after SCI

**Figure 4:**
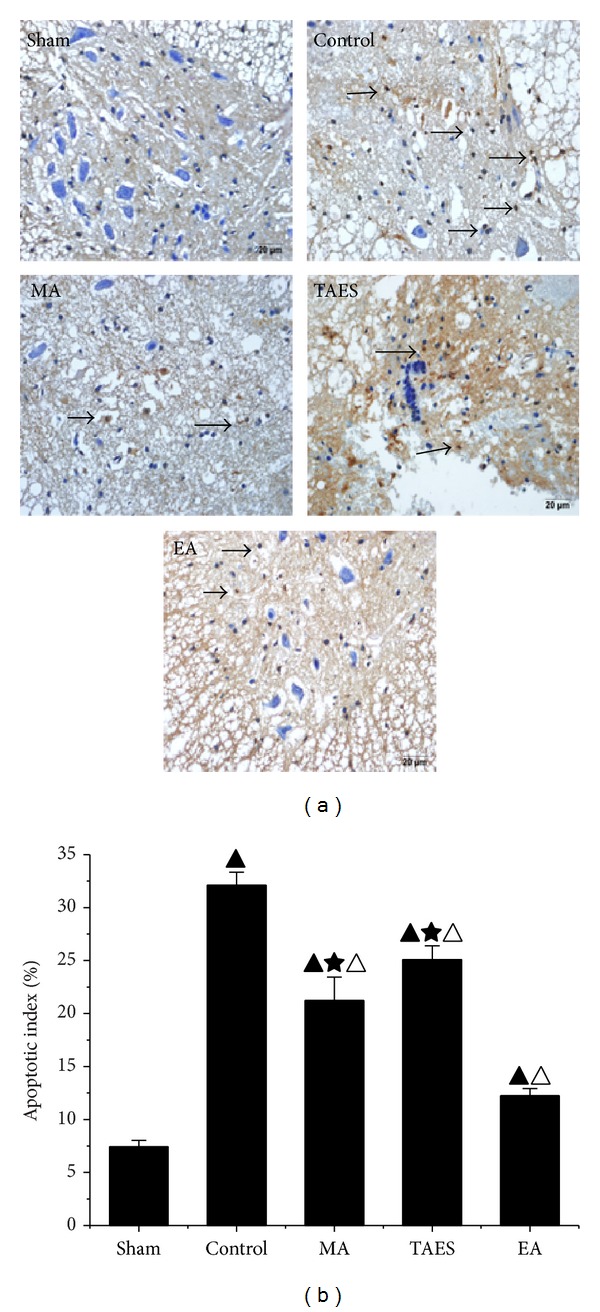
(a) TUNEL staining identified apoptotic neurons. (b) Apoptotic index. In the sham group, there were almost no TUNEL-positive cells. In the control group, positive cells were significantly increased and widely distributed in both the white and gray matters. In the control group, neurons shrank and exhibited abnormal morphology with condensed chromatin, introverted nuclear membranes, and increased apoptotic bodies (dash-line arrow). Neurons in the EA, MA, and TAES groups, however, showed less condensed chromatin and clear nuclear membranes. EA group, however, showed significantly decreased brown-positive cells compared with that of the control group (data are presented as mean ± SD, ^▲^ *P* < 0.05, versus sham group; ^△^ *P* < 0.05, versus control group; ^★^ *P* < 0.05, versus EA group). *N* = 8 animals per group.

Neurons from the sham group were all stained blue and almost no TUNEL-positive cells (brown staining) were found, whereas, in the control group, the TUNEL-positive cells were widely distributed in both the white and gray matter, with the majority found in the white matter. The nuclei of the TUNEL-positive cells were dark brown while the cytoplasm was much lighter. Moreover, some TUNEL-positive cells were found to be smaller in size with dark-brown dots. In the groups treated with EA, MA, or TAES stimulated at DU26 and DU16, the quantities of TUNEL-positive cells were dramatically decreased, which was consistent with less damage to the spinal cord structure (Figure 4(a)). This result was indicative of a protective effect of EA, MA, and TAES on neuronal apoptosis. Quantitative data of this staining was shown in Figure 4(b). EA group showed a significant decrease in the apoptosis compared with MA and TAES groups (*P* < 0.05), while no difference between MA and TAES groups (*P* > 0.05).

### 3.3. Antioxidation of Electrical, Manual Acupuncture and Transcutaneous Acupoint Electric Stimulation

**Figure 5:**
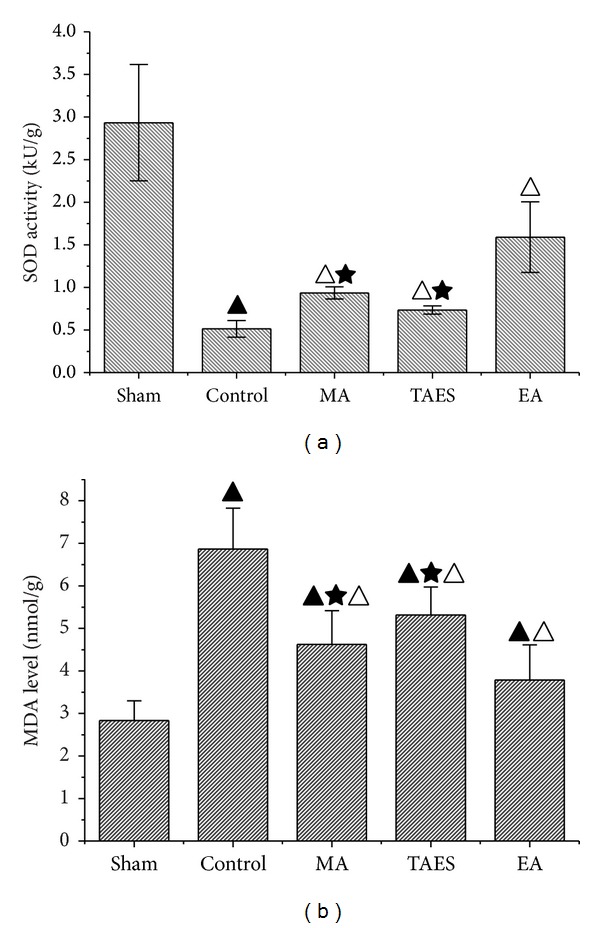
SOD and MDA level in rat spinal cord tissue. (a) Effects of EA, MA, and TAES on SOD activity at 8.5 hours of postsurgery. (b) Effects of EA, MA, and TAES on MDA level at 8.5 hours of postsurgery (data are presented as mean ± SD, ^▲^ *P* < 0.01, versus sham group; ^△^ *P* < 0.01, versus control group; ^★^ *P* < 0.05, versus EA group). *N* = 6 animals per group.

SOD activity of the spinal cord tissues from the sham group was 2.28 ± 0.17 kU/g. MDA level of the sham group was 2.83 ± 0.04 *μ*mol/g (Figure 5). In the control group, SCI caused a significant decrease in SOD activity (0.50 ± 0.04 kU/g, *P* < 0.01) and an increase in MDA level (6.87 ± 0.05 *μ*mol/g, *P* < 0.01). Compared with the control group, EA treatment after SCI dramatically increased the SOD activity to 1.60 ± 0.02 kU/g (*P* < 0.01) and decreased MDA level to 3.8 ± 0.02 *μ*mol/g (*P* < 0.01), and MA treatment increased the SOD activity to 0.93 ± 0.07 kU/g (*P* < 0.01) and decreased MDA level to 4.7 ± 0.09 *μ*mol/g (*P* < 0.01), while TAES treatment increased the SOD activity to 0.73 ± 0.04 kU/g (*P* < 0.01) and decreased MDA level to 5.31 ± 0.05 *μ*mol/g (*P* < 0.01). Meanwhile, compared with TAES and MA groups, EA group induced a more obvious change of SOD and MDA (*P* < 0.05).

### 3.4. Electrical, Manual Acupuncture and Transcutaneous Acupoint Electric Stimulation Differentially Regulate Protein Expression of TNF-*α*, IL-1*β*, and IL-6 after SCI

**Figure 6:**
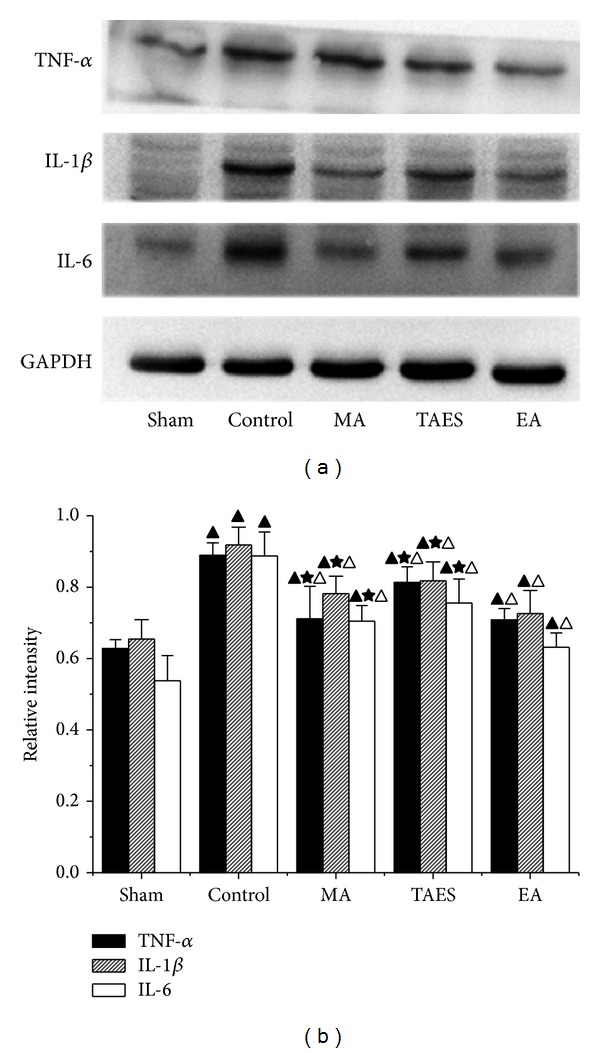
Effects of EA, MA, and TAES on the expression of inflammatory mediators. Western blots showing expression of *β*-actin, IL-1*β*, IL-6, and TNF-*α* in the spinal cords of animals at 8.5 h of postinjury (data are presented as mean ± SD, ^▲^ *P* < 0.01, versus sham group; ^△^ *P* < 0.05, versus control group; ^★^ *P* < 0.05, versus EA group). *N* = 8 animals per group.

The protein expression of TNF-*α*, IL-1*β*, and IL-6 was quantified using Western blot analysis. As shown in Figure 6(a), a basal level of TNF-*α*, IL-1*β*, and IL-6 expression was detected in the spinal cords of the sham group. Nine hours after SCI, TNF-*α*, IL-1*β*, and IL-6 expression was significantly reduced in the spinal cords of the rats with SCI. Treatment with EA, MA, or TAES significantly blunted the SCI-induced activation of TNF-*α*, IL-1*β*, and IL-6 expression (*P* < 0.05). As shown in Figure 6(b), the integrated density values (IDV) of TNF-*α* with *β*-actin in the sham group, control group, MA group, TAES group, and EA group were 0.63 ± 0.02, 0.89 ± 0.03, 0.79 ± 0.09, 0.81 ± 0.04, and 0.70 ± 0.03, respectively, at 8.5 h after SCI, and the IDVs of IL-1*β* with *β*-actin were 0.65 ± 0.05, 0.92 ± 0.04, 0.78 ± 0.05, 0.82 ± 0.05, and 0.73 ± 0.06, respectively, and the IDVs of IL-6 with *β*-actin were 0.54 ± 0.07, 0.89 ± 0.07, 0.70 ± 0.04, 0.75 ± 0.06, and 0.63 ± 0.04, respectively. IDVs of TNF-*α*, IL-1*β*, and IL-6 protein expression in the EA group were lower than MA or TAES group (*P* < 0.05).

### 3.5. Electrical, Manual Acupuncture and Transcutaneous Acupoint Electric Stimulation Differentially Regulate Gene Expression of TNF-*α*, IL-1*β*, and IL-6 mRNA after SCI

**Figure 7:**
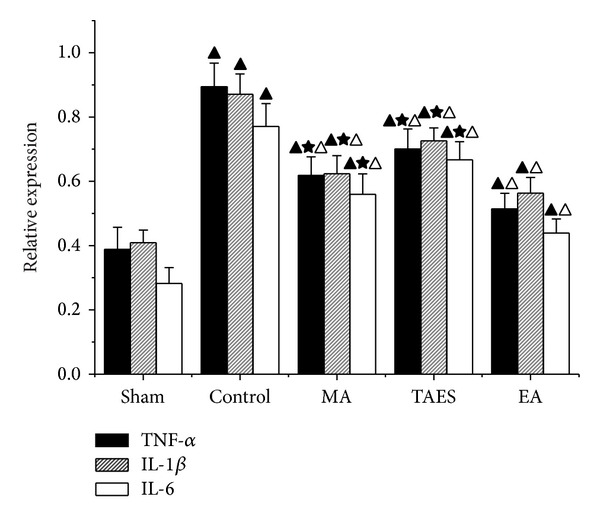
RT-PCR data of IL-1*β*, IL-6, and TNF-*α* mRNA expression levels from injured spinal cords in five groups (data are presented as mean ± SD, ^▲^ *P* < 0.01, versus sham group; ^△^ *P* < 0.01, versus control group; ^★^ *P* < 0.05, versus EA group). *N* = 8 animals per group.

To test whether treatment with EA, MA, or TAES influenced the inflammatory process through the activation and expression of proinflammatory cytokines, we analyzed the levels of TNF-*α*, IL-1*β*, and IL-6 mRNA in the spinal cord tissue following SCI. The amount of TNF-*α*, IL-1*β*, and IL-6 cytokines was 0.89 ± 0.07, 0.87 ± 0.06, and 0.77 ± 0.07, respectively, in the spinal cord tissue samples, which was found to be a substantial increase (*P* < 0.01) at 8 h following SCI (Figure 7). The relative expressions of TNF-*α*, IL-1*β*, and IL-6 mRNA of the EA group were 0.51 ± 0.04, 0.56 ± 0.05, and 0.44 ± 0.04, respectively, MA group were 0.61 ± 0.05, 0.62 ± 0.06, and 0.56 ± 0.06, respectively, and TAES group were 0.70 ± 0.06, 0.73 ± 0.04, and 0.68 ± 0.05, showing a reduction of the levels of TNF-*α*, IL-1*β*, and IL-6 mRNA compared to the control group (*P* < 0.05). Meanwhile the EA group had a lower relative expression compared with MA group and TAES group (*P* < 0.05).

## 4. Discussion

In addition to the damage at the site of the spinal cord injury, secondary pathological changes occur in the following order: edema, ischemia, calcium overload, lipid peroxidation, microcirculation obstruction, and apoptosis [17, 18]. This inflammatory response is the key point of successful therapy [19, 20]. SCI induced apoptotic cell death of neurons and oligodendrocytes has been known to cause progressive degeneration of the spinal cord, leading to permanent functional deficits [21]. Necrotic neurons, reactive glial cells, and endothelium all contribute to the production of inflammatory factors, including IL-1*β*, IL-6, and TNF-*α* [22]. These cytokines trigger activation of the endothelium which produces cellular adhesive factors mediating the adhesion between leukocyte and endothelium, thus inducing the infiltration of leukocytes in the injury site. Leukocyte infiltration breaks down the blood-spinal cord barrier and further exacerbates the traumatic injury. In our experiment, both real-time PCR and Western blot showed significant increases of IL-1*β*, IL-6, and TNF-*α* in the spinal cord after injury, which directly correlated with the extent of the spinal cord injury. This indicates that upregulation of cytokines is involved in SCI.

Previous studies have shown that EA at DU26 and DU16 increased cerebral blood flow and reduced ischemic brain injury, which resulted in the attenuation neurological deficits, infarct volume, and mortality in animal models exposed to ischemic insults [23, 24]. In addition, Choi et al. also reported that DU26 was identified as a neuroprotective acupoint after SCI [14]. However, there were no studies of these two acupoints simultaneously applied to SCI.

In 1920, it was firstly reported that electric fields played a role in nerve growth and reduction of injured nerve degeneration. Electrical stimulation which went through the spinal cord could promote the proliferation and differentiation of endogenous neural stem cells and enhance the reparative ability of nervous tissue [25–27]. It serves to generate a series of impulses passing through the spinal cord to the appropriate peripheral nerves despite a spinal cord lesion. The stimulation pulse is regulated by the following parameters: amplitude (magnitude of current), duration, frequency, wave form, and duty cycle. As an alternative strategy, electrical stimulation to needles inserted into traditional acupuncture points or simply application of electrical stimulation via electroconductive pads on the skin at these acupoints is applied in clinic. TAES avoids the use of needles and instead delivers a mild electric current at traditional acupoints which is accepted by the patients. Three modalities of acupuncture can be clinically effective, but the underlining mechanisms may be different.

But with the development of medical technology, there are more and more patients undergoing pacemaker implantation, metal stent implantation, which means EA or TAES should be limited at local acupoints. In the MA group, the fundamental manipulation twirling method is adopted. MA produces strong* deqi* sensations of fullness, heaviness, and soreness [28]. The* deqi* sensation is considered to be related to the clinical efficacy in traditional Chinese medicine. Other factors may also influence the outcome of MA, including the quantity and placement of needles, the depth of needle insertion, and the frequency of stimulation. The differential effects of manual and electrical acupuncture stimulation in this study could reflect differences in stimulation duration [29]. To our knowledge, EA, MA, and TAES stimulation have not been compared directly utilizing a SCI rat model before.

The rats that were subjected to a spinal cord contusion injury induced by NYU Impactor (10 g × 25 mm) in our study were evaluated with no observable movement of the hindlimbs or slight movement of one to two joints within 8 h of postoperation. The results matched those from the previous test [30].

Our results showed that the amounts of TUNEL-positive neurons in the spinal lamina II in EA, MA, or TAES applied at DU26 and DU16 were less than control group indicated partially restored function of neurons. Our data also suggested that all the three different modalities of acupuncture might promote the recovery of injury lesion and reverse the decrease of SOD activity and increase of MDA level at varied degree caused by SCI, which suggested an antioxidative role in response to the injury. In addition, the three different modalities of treatment suppressed immunoreactivity and expression of inflammatory cytokines including TNF-*α*, IL-1*β*, and IL-6 after SCI, which suggested an anti-inflammatory effect.

Our results suggested that, compared with MA group and TAES group, EA group could significantly promote the recovery of neuronal function. EA stimulating decreased SOD activity and increased MDA level, as well as deduced expression of inflammatory cytokines including TNF-*α*, IL-1*β*, and IL-6 compared to the other two types of treatments. We hypothesized that, in EA group, electrical pulses were delivered on the needles inserted into the acupoints, and in TAES group, only mild electric pulses were delivered on the skin of the acupoints. EA has been shown to be more effective than MA and TAES. The recent study reported that EA had a better analgesic effect than MA, while a sustained effect was better produced by MA [31]. Jiang et al. also reported that different brain mechanisms might be recruited in the three different acupuncture modalities [32]. It was indicated that the three different types of acupuncture treatment might have varied treatment effects in different extent. However, the physiological mechanisms of acupuncture are not fully understood. Therefore, further studies are required to support this speculation.

## 5. Conclusion

In summary, our data showed that EA, MA, and TAES could improve functional recovery by reducing apoptotic cell death after SCI. Meanwhile, the neuroprotective effects of the above three modalities of treatments might be mediated in part by antioxidation, anti-inflammation, and antiapoptosis effects following injury. In addition, the present study suggested that stimulating DU26 and DU16, especially with electroacupuncture, was an effective therapeutic strategy in acute spinal cord injury.
